# Large-Scale Pathway-Based Analysis of Bladder Cancer Genome-Wide Association Data from Five Studies of European Background

**DOI:** 10.1371/journal.pone.0029396

**Published:** 2012-01-04

**Authors:** Idan Menashe, Jonine D. Figueroa, Montserrat Garcia-Closas, Nilanjan Chatterjee, Nuria Malats, Antoni Picornell, Dennis Maeder, Qi Yang, Ludmila Prokunina-Olsson, Zhaoming Wang, Francisco X. Real, Kevin B. Jacobs, Dalsu Baris, Michael Thun, Demetrius Albanes, Mark P. Purdue, Manolis Kogevinas, Amy Hutchinson, Yi-Ping Fu, Wei Tang, Laurie Burdette, Adonina Tardón, Consol Serra, Alfredo Carrato, Reina García-Closas, Josep Lloreta, Alison Johnson, Molly Schwenn, Alan Schned, Gerald Andriole, Amanda Black, Eric J. Jacobs, Ryan W. Diver, Susan M. Gapstur, Stephanie J. Weinstein, Jarmo Virtamo, Neil E. Caporaso, Maria Teresa Landi, Joseph F. Fraumeni, Stephen J. Chanock, Debra T. Silverman, Nathaniel Rothman

**Affiliations:** 1 Division of Cancer Epidemiology and Genetics, National Cancer Institute, Bethesda, Maryland, United States of America; 2 Institute for Cancer Research, Surrey, United Kingdom; 3 Spanish National Cancer Research Centre, Madrid, Spain; 4 Core Genotype Facility, SAIC-Frederick, Inc., National Cancer Institute-Frederick, Frederick, Maryland, United States of America; 5 Departament de Ciències Experimentals i de la Salut, Universitat Pompeu Fabra, Barcelona, Spain; 6 Epidemiology Research Program, American Cancer Society, Atlanta, Georgia, United States of America; 7 Centre for Research in Environmental Epidemiology (CREAL), Barcelona, Spain; 8 Municipal Institute of Medical Research, Barcelona, Spain; 9 CIBER Epidemiología y Salud Pública (CIBERESP), Barcelona, Spain; 10 National School of Public Health, Athens, Greece; 11 Universitat Pompeu Fabra, Barcelona, Spain; 12 Ramón y Cajal University Hospital, Madrid, Spain; 13 Unidad de Investigación, Hospital Universitario de Canarias, La Laguna, Spain; 14 Hospital del Mar-Institut Municipal d'Investigació Mèdica (IMIM), Universitat Pompeu Fabra, Barcelona, Spain; 15 Vermont Cancer Registry, Burlington, Vermont, United States of America; 16 Maine Cancer Registry, Augusta, Maine, United States of America; 17 Dartmouth Medical School, Hanover, New Hampshire, United States of America; 18 Department of Urology, Washington University School of Medicine, St. Louis, Missouri, United States of America; 19 National Institute for Health and Welfare, Helsinki, Finland; Vanderbilt University Medical Center, United States of America

## Abstract

Pathway analysis of genome-wide association studies (GWAS) offer a unique opportunity to collectively evaluate genetic variants with effects that are too small to be detected individually. We applied a pathway analysis to a bladder cancer GWAS containing data from 3,532 cases and 5,120 controls of European background (n = 5 studies). Thirteen hundred and ninety-nine pathways were drawn from five publicly available resources (Biocarta, Kegg, NCI-PID, HumanCyc, and Reactome), and we constructed 22 additional candidate pathways previously hypothesized to be related to bladder cancer. In total, 1421 pathways, 5647 genes and ∼90,000 SNPs were included in our study. Logistic regression model adjusting for age, sex, study, DNA source, and smoking status was used to assess the marginal trend effect of SNPs on bladder cancer risk. Two complementary pathway-based methods (gene-set enrichment analysis [GSEA], and adapted rank-truncated product [ARTP]) were used to assess the enrichment of association signals within each pathway. Eighteen pathways were detected by either GSEA or ARTP at *P*≤0.01. To minimize false positives, we used the *I^2^* statistic to identify SNPs displaying heterogeneous effects across the five studies. After removing these SNPs, seven pathways (‘Aromatic amine metabolism’ [*P_GSEA_* = 0.0100, *P_ARTP_* = 0.0020], ‘NAD biosynthesis’ [*P_GSEA_* = 0.0018, *P_ARTP_* = 0.0086], ‘NAD salvage’ [*P_ARTP_* = 0.0068], ‘Clathrin derived vesicle budding’ [*P_ARTP_* = 0.0018], ‘Lysosome vesicle biogenesis’ [*P_GSEA_* = 0.0023, *P_ARTP_*<0.00012], ’Retrograde neurotrophin signaling’ [*P_GSEA_* = 0.00840], and ‘Mitotic metaphase/anaphase transition’ [*P_GSEA_* = 0.0040]) remained. These pathways seem to belong to three fundamental cellular processes (metabolic detoxification, mitosis, and clathrin-mediated vesicles). Identification of the aromatic amine metabolism pathway provides support for the ability of this approach to identify pathways with established relevance to bladder carcinogenesis.

## Introduction

Genome-wide association studies (GWAS) have served as a useful tool to identify common genetic variants associated with various complex traits [1]. As expected, each variant explains a tiny portion of the heritable component of their associated phenotypes [2], [3]. Recently, Park and colleagues estimated that some proportion of the ‘missing heritability’ may reside in additional common low-penetrance susceptibility variants that can be discovered in larger GWAS [4]. In principle, other methods could complement the primary single-locus tests of GWAS in identifying additional susceptibility loci. One such approach is pathway (gene-set) analysis [5], [6], which examines whether association signals of a collection of functionally related loci (typically genes) consistently deviate from what is expected by chance. This approach may suggest new candidate susceptibility loci and possibly provide insights into the mechanisms underlying complex traits. Pathway-based analyses have been applied to GWAS of complex diseases, including multiple sclerosis [7], type-2 diabetes [8], [9], Crohn's disease [10], [11], Parkinson's disease [12], [13], colon [14] and breast [15] cancers.

Bladder cancer is the fourth most common malignancy among men in the western world [16]. Epidemiological studies have shown that exposure to aromatic amines (AAs) from tobacco smoking or occupation is strongly associated with bladder cancer risk [16], [17], [18], [19]. Additionally, genetic studies have demonstrated that functional polymorphisms in two genes involved in carcinogen metabolism (N-acetyltransferase 2 [*NAT2*] and glutathione S-transferase M1 [GSTM1]) are associated with bladder cancer risk [20], [21]. Notably, the risk of bladder cancer associated with NAT2 slow acetylation genotype is restricted to smokers [20], [22]. Recently, a series of GWAS have identified previously unknown susceptibility loci for bladder cancer, with the prospects of more to be discovered [22], [23], [24], [25]. To identify additional regions that harbor plausible candidate genes and shed further light on genetic basis of this disease, we applied pathway analysis to the first stage of the NCI's CGEMS bladder cancer GWAS containing 3,532 cases and 5,120 controls [22]. We report here seven pathways implicated in diverse carcinogenic processes to be enriched with bladder cancer susceptibility loci.

## Materials and Methods

### Study population

We applied our analyses to primary scan data of 591,637 SNPs from NCI's bladder cancer GWAS containing 3,532 cases and 5,120 controls of European ancestry from five studies (Spanish Bladder Cancer Study [SBCS], New England, Maine and Vermont Bladder Cancer Study [NEBCS-ME/VT], Alpha-Tocopherol, Beta-Carotene Cancer Prevention Study [ATBC], the American Cancer Society Cancer Prevention Study II Nutrition Cohort [CPS-II], and the Prostate, Lung, Colorectal and Ovarian Cancer Screening Trial [PLCO]) [22].

### Pathway data construction

We collected gene-sets from five publicly available pathway resources: BioCarta [26], Kyoto Encyclopedia of Genes and Genomes (KEGG) [27], NCI's Pathway Interaction Database (PID) [28], Reactome [29], and Encyclopedia of Homo sapiens Genes and Metabolism (HumanCyc) [30]. Inclusion criteria of pathways for analysis were those containing 5–100 genes to avoid testing too narrowly- or too broadly- defined functional categories. In addition, we constructed 22 candidate pathways (Table S2) based on known bladder cancer risk factors and general carcinogenic processes [31], [32], [33] which were not represented in the public databases above. Specifically, selection of genes was determined through 1) biochemical data for the detoxification of aromatic amines [34], [35]; 2) Ingenuity pathway lists [36]; and 3) Gene ontology lists [37].

To explore the similarity between pathways in our database, we assessed the percentage of overlapping genes between each two pathways (A and B) as:
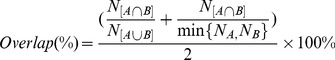
(1)where *N_A_* and *N_B_* are the number of genes within pathways A and B.

SNPs from the first stage of the NCI bladder cancer GWAS [22] were mapped to genes in these pathways if they were located in a region encompassing 20 kb 5′ upstream and 10 kb 3′ downstream from the genes' coding regions (NCBI's human genome build 36.3). These gene's boundaries were selected attempting to capture most of the gene's coding and regulatory variants [38] as well as minimizing the overlap between genes. Overall, 1,422 pathways containing 5,647genes (24.3±21.7 [mean ± SD] genes per pathway) and ∼92,000 SNPs were included in our database. A complete list of the studied pathways is available in Table S1.

### Statistical analysis

SNPs with MAF<1% among controls were excluded from the analysis. We fitted logistic regression models adjusted for age, sex, study center, DNA source (buccal/blood), and smoking status (current/former/never/occasional), to assess the marginal effect of each SNP (1 degree of freedom trend test) on the risk of bladder cancer, as previously described [22]. For each gene *G_j_* (*j* = 1, …, *N*, where *N* is the total number of genes in our dataset), the SNP with the lowest p-value among all SNPs that were mapped to its region was selected to represent the gene in the pathway analysis. We used two approaches to test for overrepresentation of association signals within pathways in our database:

- Gene-set enrichment analysis (GSEA; [12]): In this approach, the −log10 of the p-value of each gene's best SNP was used as the gene's test statistics (*r_j_* = −log10(*p_j_*). Then, a weighted Kolmogorov-Smirnov procedure was used to assess for overrepresentation of gene's statistics Enrichment Score (*ES*) within each pathway (*S*) [15].
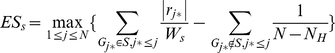
(2)where, 
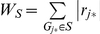
 and *N_H_* is the number of genes in a pathway.
  The statistical significance of *ES_S_* was empirically evaluated using 10,000 permutations (permuting the genotype data between individuals and keeping the LD between SNPs intact).
- Adaptive Rank-Truncated Product (ARTP; [39]): In this approach the genes' best SNP p-values (*p_j_*) in each pathway were ordered from lowest to highest. Then, the mathematical product was computed for all possible sets of *p_(j)_* such that
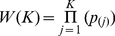
(3)with *K*, 1≤*K*≤L, being all possible integers (the truncation points) between 1 and L, with *L* being the number of genes in a pathway. In words, *W(K)* is simply the product of the K smallest *P*-values in a pathway. Next, we used the *minP* statistics [40], [41] to evaluated what is the *K* truncation point where the *W(K)* get the most statistically significant value.
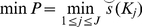
(4)where 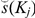 be the estimated P-value for *W(K_j_), K_1_≤…≤K*.
  We then used two-level permutation procedure (10, 000 permutations, permuting the genotype data between individuals and keeping the LD structure between SNPs intact) to estimate 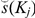, and to adjust for multiple testing over the different truncation points used.

Using both the GSEA and ARTP methods that employ different approaches to assess the enrichment of gene-based signals within predefined gene-sets may facilitate capturing a broader range of candidate pathways for bladder cancer susceptibility.

Finally, we calculated a false discovery rate (*FDR*) to assess the proportion of expected false positive findings in the GSEA and ARTP analyses. In short, we normalized the GSEA and ARTP statistics for each pathway (*NSs_(GSEA)_* and *NSs_(ARTP)_* respectively) based on the mean and standard deviation of the corresponding permutation data [12]. This procedure allows a direct comparison of pathways with different sizes and gene compositions. Then, we used these normalized statistics to calculate the FDR as:
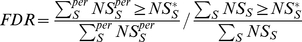
(5)


### Genetic heterogeneity analysis

To minimize false positives, we estimated the I-squared statistic (*I^2^*) [42] to identify SNPs displaying heterogeneous effects across the five studies [ATBC, CPSII, NEBCS (ME, VT), PLCO, and SBCS]. *I^2^*describes the proportion of total variation in study estimates that is due to heterogeneity. In short, a meta-analysis was applied to every SNP belonging to one of the top pathways using the genotype frequency counts of cases and controls to estimate per-allele OR and CI's. SNPs with *I^2^ P*-values<0.2 were removed from further analyses. We evaluated the OR, CI and p values for both the meta-analysis and they were similar in both models, and did not change the interpretation of the data. These analyses were done using STATA (Version 11, STATA Corporation, College Station, TX).

## Results

Overall, there was good correlation between the results of the GSEA and the ARTP methods (r = 0.74, P<0.0001). A detailed examination of the results revealed that, on average, GSEA performed better in detecting pathways enriched with multiple weak association signals while ARTP appeared to be more powerful in detecting pathways where only few genes with relatively strong signals are dominating. Notably, the AA metabolism pathway, which contains several known bladder cancer susceptibility loci, was detected by both GSEA and ARTP methods (*P_GSEA_* = 0.0100, *P_ARTP_* = 0.0020). Therefore, we used its significance level as a reference for highlighting additional candidate susceptibility pathways. Of the 1421 pathways examined, 18 were significantly enriched with association signals at the *P*<0.01 level (Table 1). Of these, seven pathways were detected by both GSEA and ARTP, four pathways were detected only by GSEA, and seven were detected only by ARTP. After removing SNPs with heterogeneous effects across the five studies (*I^2^ P*-value<0.2), the enrichment signals remained significant (*P*<0.01) in seven pathways belonging to four cellular processes (“aromatic amine [AA] metabolism”, “Nicotinamide adenine dinucleotide [NAD] metabolism”, “Clathrin-mediated vesicles”, and “Mitosis”). For clarity, from this point forward, we will refer only to the results from the post heterogeneity analysis.

**Table 1:** Pathways enriched with bladder cancer susceptibility loci at a *P*≤0.01 level using GSEA and ARTP.

			GSEA			ARTP			Gene overlap (%)
Pathway	source	# genes^1^	# genes^2^	p-value^3^	FDR^4^	# genes^2^	p-value^3^	FDR^4^	
Aromatic amine metabolism	Self	11	(5); 1	**(0.0059); 0.0100**	(>0.5)	(9); 1	**(0.0012); 0.0020**	(0.28)	NA
NAD biosynthesis I (from aspartate)	HumanCyc	5	(4); 4	**(0.0021); 0.0018**	(>0.5)	(4); 4	**(0.0086); 0.0086**	(0.36)	44%
NAD salvage pathway II	HumanCyc	9	(5); 6	(0.0150); 0.0583	(>0.5)	(7); 8	**(0.0033); 0.0068**	(0.32)	
Clathrin derived vesicle budding	Reactome	15	(6); 6	(0.0210); 0.0189	(>0.5)	(9); 9	**(0.0018) 0.0018**	(0.35)	
Lysosome Vesicle Biogenesis	Reactome	10	(6); 7	**(0.0031); 0.0023**	(>0.5)	(7); 7	**(<0.0001); <0.0001**	(0.16)	49%
Retrograde neurotrophin signaling	Reactome	9	(4); 4	**(0.0092); 0.0084**	(>0.5)	(4) ;4	(0.0192); 0.0192	(0.41)	
Mitotic Metaphase/Anaphase Transition	Reactome	8	(3); 3	**(0.0043); 0.0040**	(>0.5)	(3); 3	(0.0187); 0.0187	(0.43)	55%
Mitotic Prometaphase	Reactome	80	(12); 12	(0.0955); 0.2567	(>0.5)	(13); 12	**(0.0095)**; 0.0346	(0.37)	
Control of skeletal myogenesis by hdac and calcium/calmodulin-dependent kinase (camk)	BioCarta	21	(11); 10	(0.1216); 0.2322	(>0.5)	(7); 3	**(0.0040)**; 0.0617	(0.29)	12%
B cell receptor signaling pathway	KEGG	75	(29); 28	(0.1121); 0.1931	(>0.5)	(10); 9	**(0.0059)**; 0.0244	(0.38)	
Syndecan-1-mediated signaling events	PID	15	(12); 9	**(0.0014)**; 0.0388	(>0.5)	(12); 11	**(0.0092)**; 0.1666	(0.43)	18%
Syndecan-2-mediated signaling events	PID	31	(19); 16	**(0.0048)**; 0.0559	(>0.5)	(31); 31	**(0.0078)**; 0.1404	(0.42)	
TGF-beta signaling pathway	KEGG	85	(41); 36	**(0.0090)**; 0.0988	(>0.5)	(57); 57	(0.0251); 0.2196	(>0.5)	NA
Activated AMPK stimulates fatty-acid oxidation in muscle	Reactome	8	(4); 3	(0.0434); 0.2470	(>0.5)	(8); 8	**(0.0017)**; 0.0454	(0.41)	
AMPK inhibits chREBP transcriptional activity	Reactome	5	(3); 2	**(0.0010)**; 0.0411	(>0.5)	(3); 2	**(0.0014)**; 0.0465	(0.33)	39%
Reversal of insulin resistance by leptin	BioCarta	10	(5); 7	(0.0170); 0.6432	(>0.5)	(10); 2	**(0.0028)**; 0.1635	(0.37)	
Maturity onset diabetes of the young	KEGG	25	(12); 11	**(0.0067)**; 0.0308	(>0.5)	(12); 16	(0.0390); 0.1908	(>0.5)	NA
Metabolism of polyamines	Reactome	12	(6); 4	**(0.0055)**; 0.0460	(>0.5)	(7); 5	**(0.0040)**; 0.0657	(0.32)	NA

Results of the top ranked pathways (*P*<0.01) using GSEA and ARTP. In parenthesis are results prior of removal SNPs displaying heterogeneous signals.

1 The number of genes in the pathway.

2 The number of genes underlying the enrichment signal in the pathway.

3 *P*-value of the enrichment score based on 10,000 permutations.

4 False-discovery rate calculated based on the normalized statistics of the permutation data to account for the variable sizes of genes and pathways.

### Aromatic amine [AA] metabolism

Table 2 displays the results for the genes in the AA pathway. The enrichment signals in this pathway were mainly driven by SNPs in the *UGT1A9* and *NAT2* genes. SNPs in these genes were identified in the primary analysis of this GWAS [22]. Removing these two genes from the pathway analyses reduced the enrichment signal in the AA metabolism pathway in both methods but still ranked it relatively high using the GSEA (*P_GSEA_* = 0.0130, *P_ARTP_* = 0.1217). Apart from *UGT1A9* and *NAT2*, five additional genes in this pathway had SNPs with significant genetic effect (*P*_trend_<0.05). These included *NAT1*, *UGT1A4*, *UGT1A6*, *NQO1* and *CYP1B1*.

**Table 2:** Summary of genes in the aromatic amine metabolism pathway used for pathway-based analysis of multi-study bladder cancer GWAS.

Gene	# SNPs^1^	SNP^2^	SNP^3^rank	MAF^4^	Allelic OR (95% CI)^5^	*P*-value^6^		
*UGT1A9*	72	rs11892031	1	0.08	0.77	0.68	0.87	3.6×10^−5^
*NAT2*	15	rs4646249	1	0.28	0.89	0.83	0.95	0.0013
*NAT1*	11	rs9650592	1	0.11	0.86	0.78	0.96	0.0054
*UGT1A4*	41	rs4148328	1	0.38	0.91	0.85	0.98	0.0086
*UGT1A6*	62	rs4148328	1	0.38	0.91	0.85	0.98	0.0086
*NQO1*	6	rs1437135	1	0.20	0.91	0.84	0.99	0.0275
*CYP1B1*	13	rs2855658	1	0.43	0.94	0.88	1	0.0477
*CYP1A1*	4	rs2472297	2	0.22	1.03	0.95	1.11	0.4758
*CYP1A2*	5	rs2472297	4	0.22	1.03	0.95	1.11	0.4758
*SULT1A1*	1	rs1968752	1	0.37	1.01	0.95	1.08	0.7321
*SULT1A2*	1	rs4788073	1	0.37	0.99	0.93	1.06	0.8344

1 Number of SNPs genotyped in the gene region (20 kb 5′ upstream and 10 kb 3′ downstream from the gene's coding region).

2 The SNP representing the gene in the pathway analysis after the removal of SNPs with heterogeneous effects.

3 The rank of the SNP among all SNPs in the gene's region based on their p-values.

4 Minor allele frequency among controls.

5 Per allele odds ratios +95% confidence intervals from logistic regression models adjusting for age, sex, study center, DNA source , and smoking.

6 1 d.f. trend test.

Some of the genes in the AA metabolism pathway (i.e. *CYP1A1* and *CYP1A2*; *UGT1A4*, *UGT1A6* and *UGT1A9*; *SULT1A1* and *SULT1A2*) occur on the same chromosomal locus and consequently share similar tagging SNPs. To assess the effect of this redundancy on the pathway enrichment signal, we pooled together genes with overlapping SNPs and treated them as a single genetic unit in our pathway analyses. Consequently, the number of loci included in the AA metabolism pathway was reduced to seven, (Table S2) and the corresponding enrichment signals were strengthened (*P_GSEA_* = 0.0046, *P_ARTP_* = 0.0001). Even when removing the *NAT2* and *UGT1A* regions from this gene-set, its corresponding enrichment signal remains relatively high (*P_GSEA_* = 0.024, *P_ARTP_* = 0.0921).

### NAD metabolism

Two nicotinamide adenine dinucleotide (NAD) metabolism pathways were detected in this analysis. The “NAD biogenesis I” pathway (HumanCyc) was detected by both GSEA and ARTP (*P_GSEA_* = 0.0018, *P_ARTP_* = 0.0086), and the “NAD salvage II” pathway (HumanCyc) was detected only by the ARTP method (*P_ARTP_* = 0.0068). Table 3 presents the results for the genes in these pathways. The three NMNAT genes (*NMNAT1*, *NMNAT2*, and *NMNAT3*) that are shared by both of these two pathways harbor SNPs with significant genetic effect (*P_trend_*<0.05) and therefore likely to dominate the significant enrichment signals in these pathways. Other genes displaying significant bladder cancer risk are *QPRT* in the “NAD I” pathway, and *ACP6*, *ITGB1BP3*, *ACPL2* in the “NAD II” pathway.

**Table 3:** Summary of genes in the NAD metabolism pathways used for pathway-based analysis of multi-study bladder cancer GWAS.

Pathway	Gene	# SNPs^1^	SNP^2^	SNP^3^ rank	MAF^4^	Allelic OR (95% CI)^5^			*P*-value^6^
NAD1/NAD2	NMNAT3	36	rs7636269	1	0.48	1.12	1.05	1.20	0.0004
NAD2	ACP6	16	rs1344	1	0.41	1.11	1.04	1.18	0.0017
NAD1	QPRT	7	rs3862476	1	0.07	1.19	1.04	1.35	0.0087
NAD1/NAD2	NMNAT2	36	rs4652795	1	0.38	0.92	0.86	0.98	0.0099
NAD1/NAD2	NMNAT1	8	rs1220398	1	0.14	0.89	0.81	0.98	0.0169
NAD2	ITGB1BP3	8	rs2304191	1	0.11	1.11	1.01	1.23	0.0355
NAD2	ACPL2	31	rs3210458	2	0.09	1.12	1.00	1.25	0.0421
NAD2	NUDT12	5	rs371315	1	0.28	1.07	1.00	1.15	0.0686
NAD2	NT5C3L	6	rs9907244	1	0.43	0.95	0.89	1.01	0.1094
NAD1	NADSYN1	17	rs4945007	1	0.06	1.10	0.96	1.25	0.1555
NAD2	C9orf95	19	rs7021664	1	0.08	0.94	0.83	1.06	0.3193

1 Number of SNPs genotyped in the gene region (20 kb 5′ upstream and 10 kb 3′ downstream from the gene's coding region).

2 The SNP representing the gene in the pathway analysis after the removal of SNPs with heterogeneous effects.

3 The rank of the SNP among all SNPs in the gene's region based on their p-values.

4 Minor allele frequency among controls.

5 Per allele odds ratios +95% confidence intervals from logistic regression models adjusting for age, sex, study center, DNA source , and smoking.

6 1 d.f. trend test.

### Vesicle biogenesis and budding

Three pathways involved in clathrin-dependent vesicle biogenesis and budding were detected in this analysis. The “Lysosome Vesicle Biogenesis” pathway (Reactome) showed the strongest enrichment signal among all pathways in this study, and was detected by both GSEA and ARTP (*P_GSEA_* = 0.0023, *P_ARTP_*<0.0001). The “Clathrin derived vesicle budding” pathway (Reactome) was detected only by ARTP (*P_ARTP_* = 0.0018), while the “Retrograde neurotrophin signaling” pathway (Reactome) was detected only by GSEA (*P_GSEA_* = 0.0084). Table 4 displays the results for the genes in these pathways. Three genes are shared by the three pathways: *CLTA* and *CLTC*, which encode for the light and heavy chains of clathrin respectively, and *SH3GL2* which is associated with clathrin-mediated endocytosis. The association of SNPs in these three genes with bladder cancer risk ranked them among the top four genes in these pathways.

**Table 4:** Summary of genes in the Clathrin-mediated vesicle pathways used for pathway-based analysis of multi-study bladder cancer GWAS.

Pathway	Gene	# SNPs^1^	SNP^2^	SNP^3^rank	MAF^4^	Allelic OR (95% CI)^5^			*P*-value^6^
Clathrin/Lysosome/Retrograde	CLTA	10	rs10972786	1	0.06	1.27	1.11	1.45	0.0004
Clathrin/Lysosome	ARRB1	29	rs667791	1	0.39	1.11	1.04	1.19	0.0014
Clathrin/Lysosome/Retrograde	SH3GL2	92	rs2209426	1	0.17	0.87	0.80	0.95	0.0020
Clathrin/Lysosome/Retrograde	CLTC	10	rs7224631	1	0.09	1.19	1.06	1.32	0.0023
Clathrin/Lysosome	DNAJC6	38	rs1325607	1	0.21	1.12	1.03	1.21	0.0057
Clathrin/Lysosome	HSPA8	8	rs11218950	1	0.05	0.80	0.68	0.95	0.0087
Retrograde	NGF	45	rs12760036	1	0.10	0.85	0.76	0.96	0.0096
Clathrin/Lysosome	AP1G1	7	rs9932707	1	0.45	1.07	1.00	1.14	0.0353
Clathrin	VAMP2	3	rs3202848	1	0.37	0.93	0.86	1.00	0.0572
Clathrin	VAMP8	9	rs719023	1	0.39	0.94	0.88	1.00	0.0631
Retrograde	DNAL4	7	rs738141	1	0.17	1.08	1.00	1.18	0.0645
Clathrin	SNAP23	3	rs4924682	1	0.01	1.27	0.95	1.70	0.1087
Clathrin/Lysosome	DNM2	16	rs4804528	1	0.43	0.95	0.89	1.02	0.1437
Retrograde	DNM1	13	rs13285411	1	0.12	0.93	0.84	1.03	0.1463
Clathrin/Lysosome	AP1B1	14	rs5763140	1	0.11	1.08	0.97	1.19	0.1500
Clathrin/Lysosome	ARF1	4	rs3768331	1	0.38	1.05	0.98	1.12	0.1536
Clathrin	GBF1	15	rs1057050	1	0.06	0.90	0.78	1.04	0.1673
Retrograde	NTRK1	13	rs1888861	1	0.23	0.95	0.88	1.03	0.2275
Retrograde	AP2A2	12	rs7483870	1	0.23	0.96	0.89	1.04	0.3014
Retrograde	AP2A1	9	rs2286948	1	0.36	1.03	0.96	1.10	0.3694
Clathrin	STX4	1	rs10871454	1	0.39	1.00	0.94	1.07	0.9722

1 Number of SNPs genotyped in the gene region (20 kb 5′ upstream and 10 kb 3′ downstream from the gene's coding region).

2 The SNP representing the gene in the pathway analysis after the removal of SNPs with heterogeneous effects.

3 The rank of the SNP among all SNPs in the gene's region based on their p-values.

4 Minor allele frequency among controls.

5 Per allele odds ratios +95% confidence intervals from logistic regression models adjusting for age, sex, study center, DNA source, and smoking.

6 1 d.f. trend test.

### Mitosis

The “Mitotic metaphase/anaphase transition” (Reactome) was detected by the GSEA method (*P_GSAE_* = 0.0040) and was marginally significant using ARTP (*P_ARTP_* = 0.0187). Interestingly, all eight genes in this pathway are included in the more comprehensive “Mitotic prometaphase” pathway that was detected in the initial pathway screening, but had a less significant signal after removing SNPs with heterogeneous signals (Table 1). Results for the eight genes included in the “Mitotic metaphase/anaphase transition” pathway are presented in Table 5. Three SNPs in three genes (*FBXO5*, *SMC3* and *SPC24*) were associated with significant protective effect on bladder cancer (*P_trend_*<0.05).

**Table 5:** Summary of genes in the Mitotic Metaphase/Anaphase Transition pathway used for pathway-based analysis of multi-study bladder cancer GWAS.

Gene	# SNPs^1^	SNP^2^	SNP^3^ rank	MAF^4^	Allelic OR (95% CI)^5^			*P*-value^6^
FBXO5	11	rs9479476	1	0.11	0.83	0.75	0.93	0.0010
SMC3	8	rs7918064	1	0.27	0.90	0.84	0.97	0.0073
SPC24	18	rs4804149	2	0.28	0.92	0.85	0.99	0.0202
CENPQ	7	rs4267943	1	0.36	0.94	0.87	1.01	0.0706
NDC80	15	rs13381300	1	0.07	0.91	0.80	1.04	0.1673
NUP107	7	rs11177325	1	0.31	0.95	0.89	1.02	0.1951
CENPA	4	rs2060390	1	0.26	0.98	0.91	1.06	0.6106
SMC1A	2	rs1264013	1	0.42	1.00	0.95	1.05	0.9876

1 Number of SNPs genotyped in the gene region (20 kb 5′ upstream and 10 kb 3′ downstream from the gene's coding region).

2 The SNP representing the gene in the pathway analysis after the removal of SNPs with heterogeneous effects.

3 The rank of the SNP among all SNPs in the gene's region based on their p-values.

4 Minor allele frequency among controls.

5 Per allele odds ratios +95% confidence intervals from logistic regression models adjusting for age, sex, study center, DNA source , and smoking.

6 1 d.f. trend test.

## Discussion

Our pathway-based analysis of a large bladder cancer GWAS using two complementary pathway-based methods (GSEA and ARTP) identified an overrepresentation of association signals in seven pathways (‘Aromatic amine metabolism’, ‘NAD biosynthesis’, ‘NAD salvage’, ‘Clathrin derived vesicle budding’, ‘Lysosome vesicle biogenesis’, ‘Retrograde neurotrophin signaling’, and ‘Mitotic metaphase/anaphase transition’) and suggest involvement in at least three cellular processes (metabolic detoxification, mitosis, and clathrin-mediated vesicles).

The identification of the AA metabolism pathway in this study by both GSEA and ARTP could be considered a good indication for the utility of this approach, since AA metabolism has established relevance to bladder cancer susceptibility. Interestingly, the enrichment signal in this pathway is driven by variations in the *UGT1A* gene cluster and the *NAT1*, *NAT2*, and *NQO1* genes (Table 1) that are involved in detoxification processes in the AA pathway [34], [35]. The strong enrichment signal left in this pathway even after the removal of the *UGT1A* and *NAT2* genes from the analysis indicates that other genetic variations affecting aromatic amines detoxification may contribute to bladder cancer susceptibility.

The detection of the NAD metabolism pathway may be relevant to bladder cancer susceptibility through several carcinogenic mechanisms. First, NAD homeostasis has been shown to play a role in various redox reactions that may lead to irreversible cellular damage and consequently to the initiation of malignant tumor [43]. In addition, NAD has been shown to be involved in DNA repair and telomere maintenances [44] as well as in energy production both of which are important processes in cancer development. Interestingly, NAD metabolism pathway has been implicated in a recent pathway-based analysis of colon cancer GWAS [14]. Colon and bladder cancers have been associated with NAT2 acetylation status. For bladder cancer, in which N-acetylation is a detoxification step, NAT2 slow acetylator phenotype presents a higher risk. In contrast, for heterocyclic amine-related colon cancer in which N-acetylation is negligible and O-acetylation is a carcinogen-activation step, NAT2 rapid acetylator phenotype presents a higher risk [45]. Thus, similar metabolic pathways could play diverse roles in the etiology of these two cancers.

Three clathrin-mediated vesicle pathways are also highlighted in this study. Clathrin-coated vesicles play essential role in intracellular trafficking, endocytosis, and exocytosis [46]. In this realm, it has been shown that clathrin-mediated vesicle pathways regulate the signaling and cellular localization of several growth factors [47] that are known to play a role in cancer susceptibility. Interestingly, clathrin may be also relevant to the Mitotic Metaphase/Anaphase transition pathway that was also implicated in this study. During mitosis, clathrin helps stabilizing the kinetochore fibers which are required for the proper function of the mitotic spindle [48]. Thus, the overrepresentation of association signals in two distinct pathways associated with mitosis suggest that perturbations in the mitotic process, and particularly those related to the metaphase/anaphase transition, may modify the risk of human bladder cancer.

Strengths of our study are the large sample size; the use of primary scan data from five independent studies allowing us to address consistency of effects across the different populations; and the use of two complementary pathway-based methods. A limitation of our study is the lack of pathway-based signals to reach a noteworthy FDR significance level, with only one pathway (Lysosome Vesicle Biogenesis) having an FDR value <0.2. This could be partially due to the inherent limits of the methods used, the inadequate annotation of relevant pathways in public databases, or due to weak association signals in our data. Recent analysis of bladder cancers using RNA expression data, have also highlighted enrichment of genes with similar processes as we identified in our genomic data here, including metabolic processes, which provide further plausibility that the pathways identified may be relevant to bladder cancer susceptibility [49]. Furthermore, the high rank of the AA metabolism pathway in both GSEA and ARTP support the power of these methods to highlight pathways with established relevance to bladder cancer susceptibility and may therefore similarly suggest the involvement of metabolic detoxification, mitosis and clathrin-mediated pathways in bladder carcinogenesis.

## Supporting Information

Table S1 **Details and results for all 1423 pathways included in this study.**
(XLS)

Table S2 **List of genes included in the 22 self-constructed candidate pathways.**
(XLS)
